# Toward the Quantification of a Conceptual Framework for Movement Ecology Using Circular Statistical Modeling

**DOI:** 10.1371/journal.pone.0050309

**Published:** 2012-11-30

**Authors:** Ichiro Ken Shimatani, Ken Yoda, Nobuhiro Katsumata, Katsufumi Sato

**Affiliations:** 1 The Institute of Statistical Mathematics, Tokyo, Japan; 2 Graduate School of Environmental Studies, Nagoya University, Furo, Chikusa, Nagoya, Japan; 3 International Coastal Research Center, Atmosphere and Ocean Research Institute, The University of Tokyo, Kashiwa, Chiba, Japan; Hokkaido University, Japan

## Abstract

To analyze an animal’s movement trajectory, a basic model is required that satisfies the following conditions: the model must have an ecological basis and the parameters used in the model must have ecological interpretations, a broad range of movement patterns can be explained by that model, and equations and probability distributions in the model should be mathematically tractable. Random walk models used in previous studies do not necessarily satisfy these requirements, partly because movement trajectories are often more oriented or tortuous than expected from the models. By improving the modeling for turning angles, this study aims to propose a basic movement model. On the basis of the recently developed circular auto-regressive model, we introduced a new movement model and extended its applicability to capture the asymmetric effects of external factors such as wind. The model was applied to GPS trajectories of a seabird (*Calonectris leucomelas*) to demonstrate its applicability to various movement patterns and to explain how the model parameters are ecologically interpreted under a general conceptual framework for movement ecology. Although it is based on a simple extension of a generalized linear model to circular variables, the proposed model enables us to evaluate the effects of external factors on movement separately from the animal’s internal state. For example, maximum likelihood estimates and model selection suggested that in one homing flight section, the seabird intended to fly toward the island, but misjudged its navigation and was driven off-course by strong winds, while in the subsequent flight section, the seabird reset the focal direction, navigated the flight under strong wind conditions, and succeeded in approaching the island.

## Introduction

Movement ecology is currently at the stage of unifying several paradigms that have tended to be investigated separately [1]. For large animals, recent technological developments in bio-logging science have enabled us to obtain movement trajectory data with high resolution, e.g., GPS locations at 1 s or shorter intervals [2], [3]. In contrast, statistical techniques for analyzing these spatiotemporal data have been developing slowly. Although various new movement models have been introduced [4], [5], for modeling the direction of heading, most of them essentially rely on a correlated random walk (CRW),
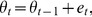
(1)where θ*_t_* is the heading direction from time *t* to *t* +1, and *e_t_* is independently and identically distributed as a circular probability distribution. Unfortunately, many real trajectories have shown movement patterns that cannot be realized by CRW [6], [7]. In particular, during a long period of movement, animals seem to have some “focal” direction (i.e., a direction that the animal intends to move toward), and CRW can rarely realize such oriented trajectories. Hence, we need to develop a basic time-series model that can flexibly cover a broad range of movement patterns. In addition, the model parameters should accompany ecological interpretations and quantify some important aspects of animal behavior; i.e., the modeling framework should be closely related to the quantification process of a conceptual framework in movement ecology. Moreover, the basic model must be mathematically tractable [4], [5], [8].

Previously proposed models have not satisfied these conditions. For example, Nams [9] introduced the following oriented movement model. Let α be the focal direction. An animal adjusts the direction of heading at every time step as follows:
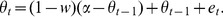
(2)


If *w* = 0, θ*_t_* = *α*+*e_t_*, thus, the animal tries to change the direction of heading toward α, with a stochastic error (*e_t_*). If *w* = 1, equation (2) reduces to the CRW (equation (1)). If *w* is close to zero, a strong adjustment operates, and model (2) continuously covers from CRW to oriented movements. However, this model contains unreasonable properties. First, equation (2) is not continuous at α – π and is therefore not mathematically so tractable (see Appendix S1). Second, previous studies have used symmetric probability distributions for the stochastic term (*e_t_*), although actual GPS trajectories often contain asymmetry, e.g., when a bird flies under windy conditions (see Figures 5 and 7, below).

The circular auto-regressive time-series model introduced in Kato [10] and the asymmetric circular probability distribution introduced in Kato and Jones [11] can solve these problems. In addition, the applicability of these circular statistical modeling is not limited to oriented trajectories but also to tortuous movements. This study aimed to formulate a basic movement model and to relate the modeling framework with the conceptual framework for movement ecology, as a quantitative methodology.

This paper begins with the formulation of the movement models, followed by a discussion regarding their correspondence to the conceptual framework summarized by Nathan et al. [1]. Using examples of the models being applied to the GPS trajectories of a seabird, the paper explains how the model parameters can be ecologically interpreted and how animal movements can be quantitatively evaluated. The final section discusses unsolved issues and future directions.

## Materials and Methods

### Movement Model

Here, we denote the trajectory of an animal movement by **x** *_t_* = (**x**_0_, **x**_1_, …, **x**_n_) where **x** *_t_* = (*x*_1*t*_, *x*_2*t*_) is the location (*x*–*y* coordinate) at time *t* (*t* = 0, 1, …, *n*). The direction of heading from time *t* to *t* +1 is given by 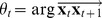 (throughout the paper, directions are as 0 = east, π/2 = north, π = west, and 3π/2 = south), and the speed is given by 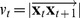 (*t* = 0, 1, …, *n* –1). We assumed that locations are acquired at regular intervals and that the direction of heading and speed was constant within each interval.

When the model was applied to real data, we used the notation **X** *_t_* for observed locations at time *t*, 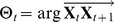 for the observed direction of heading, and 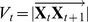 for the observed speed (actually, Θ*_t_* and *V_t_* are “the observed orientation” and “the observed step length,” but for simplicity, the above terminologies are used in this paper).

The modeling of an animal’s movement is given by
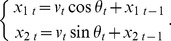
(3)


Here, we formulated the direction of heading θ*_t_* first, then the speed *v_t_* and then considered other specific details after the description of the real GPS data.

### Heading Model

**Figure 1:**
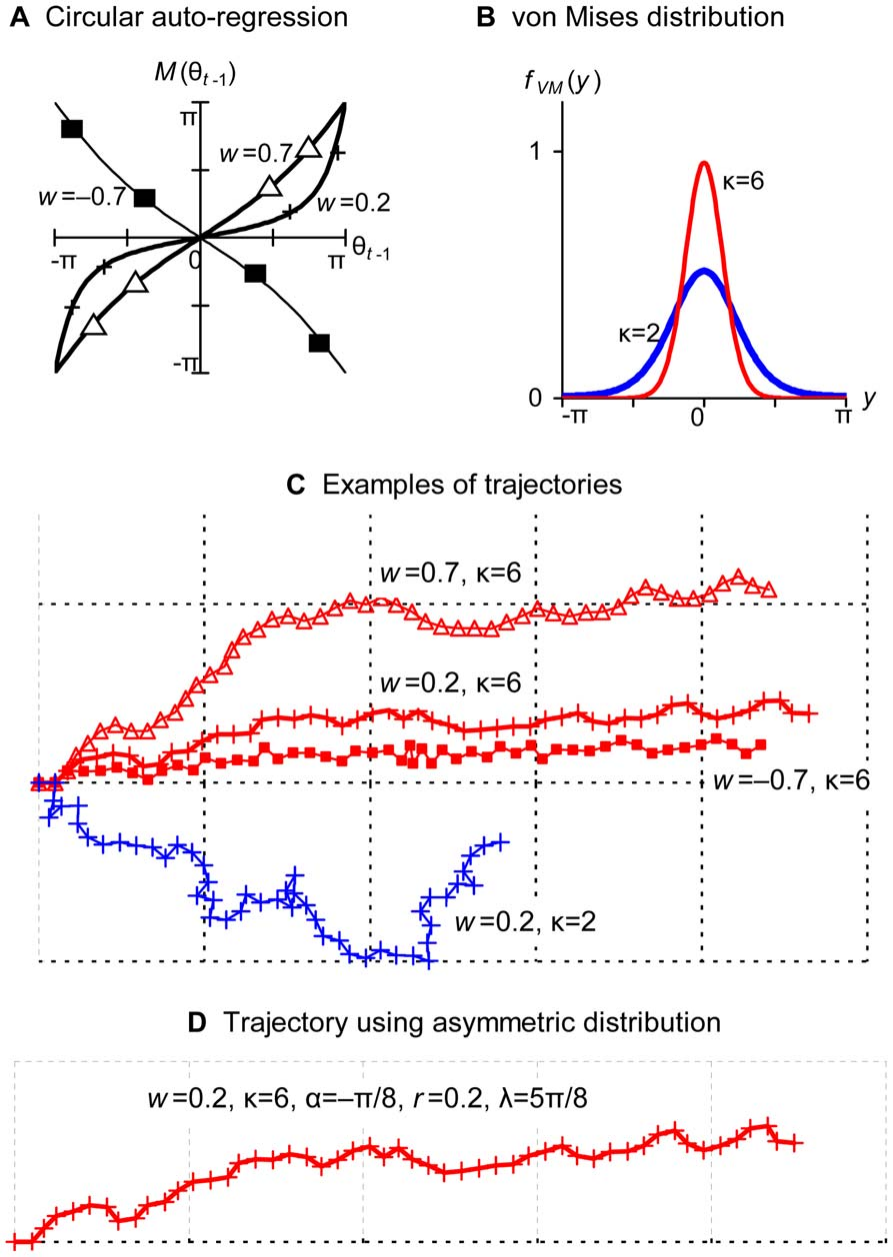
Examples of trajectories simulated by the movement model. (A) Examples of the transformation θ*_t_* = *M*(θ*_t_*_–1_; α, *w*) (equation (5), α = 0). (B) Examples of the probability density functions of the von Mises distribution (equation (7), μ = 0). (C) Examples of trajectories produced by equation (3) using the heading model (4) with the transformation in (A) and the von Mises distribution in (B). (D) An example of trajectory produced by equation (3) using the heading model (12). In (C) and (D), speeds were fixed at 1, the grid unit is 10, and the first 50 steps are shown. The same random samples from the von Mises distribution of κ = 6 were used for the four red trajectories.

For modeling the direction of heading, we applied Kato’s circular auto-regressive time-series model (C-AR) [10] given by
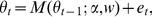
(4)where
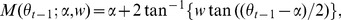
(5)w is the regression coefficient, α is the focal direction, and 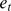 is a random angle that is independently and identically distributed (i.i.d.) as a circular distribution (e.g., equations (7) and (8) below). Equation (5) gives a smooth correspondence from the previous direction of heading (θt−1) to the subsequent θt (Figure 1A) and resolves the discontinuity of model (2).

Kato’s circular auto-regressive model is a modification of the circular–circular regression in Downs and Mardia [12] defined as
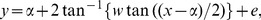
(6)where *x* and *y* are circular variables (–π ≤ *x*, *y*<π), *w* is the regression coefficient, and α is the focal direction. This is an example of a generalized linear model (GLM), where 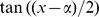 is used as a link function for transforming circular variables to linear variables. In fact, substituting 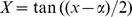 and 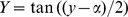 into the linear regression equation *Y = wX*, we obtained 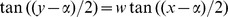, which is equivalent to equation (6).

Because the link function 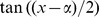 has an inflexion point, the regression line is sigmoidal, which is commonly observed in GLMs (e.g., logistic regression). A key difference is that the circular–circular regression uses the link function twice because both independent and dependent variables are circular. Consequently, dependent variables tend to be attracted to the focal direction (α), and the attraction becomes stronger if *w* is close to 0 (Figure 1A).

The attraction toward α could be considered inappropriate as an extension of “linear” regression; however, if the model is extended to a circular auto-regressive time-series model (equation (4)) and applied to animal movements, the direction of heading is adjusted toward the focal direction at every time step. This is a desirable property for the oriented movement of an animal, as demonstrated in Figure 1C.

### Circular Probability Distribution

The commonly used circular probability distributions and their probability density functions are [13]

VM(μ, κ) = von Mises distribution,
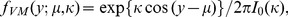
(7)where κ >0 and *I*_0_(κ) is the modified Bessel function of the first kind and zero order, and

WC(μ, *r*) = wrapped Cauchy distribution,
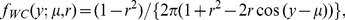
(8)where 0≤ *r* <1.

Both probability density functions reach a maximum at μ and are symmetric with respect to *y* = μ. In model (4), we set μ = 0. κ in the von Mises distribution, and *r* in the wrapped Cauchy distribution determines the degree of concentration around μ. Greater values of κ and *r* produce more strictly concentrated von Mises and wrapped Cauchy distributions, respectively (Figure 1B).

### Kato–Jones Distribution

If observed directions of heading are not distributed symmetrically (such examples can be seen in Figures 5 and 7), the model using the von Mises distribution or wrapped Cauchy distribution is not appropriate. This study used the Kato–Jones distribution [11]. The probability density function is given by

KJ(μ, κ, *r*, ν) = Kato–Jones distribution,
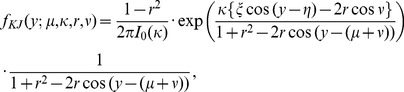
(9)where 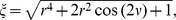 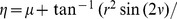 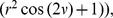 –π ≤ μ, ν<π, κ >0, 0≤ *r* <1.

This complex distribution is produced by transforming symmetric circular random variables *X* ∼ VM(0, κ) to (∼ means “distributed as”)
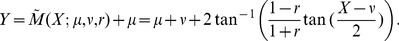
(10)


Kato and Jones [11] showed that the circular probability density function of *Y* is given by equation (9).

The transformation (10) is essentially the same as transformation (6); circular variable *X* is first attracted to ν and is then shifted (rotated) by angle μ. Alternatively, we may interpret the Kato–Jones distribution as random variables 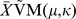 are transformed by.
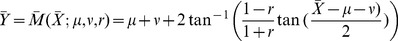
(10<?ENTCHAR rsquo?>)


**Figure 2:**
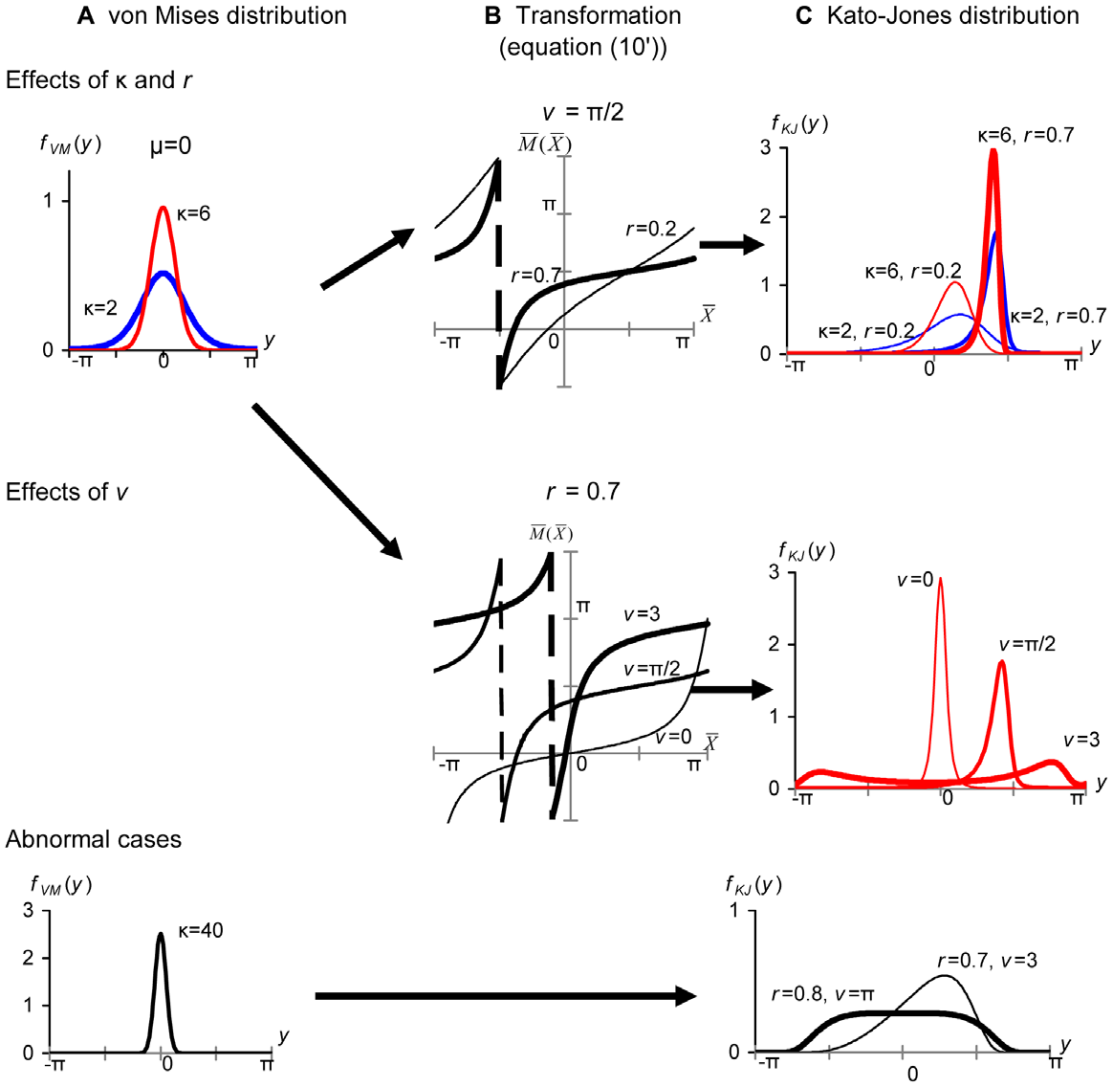
The method of generating the Kato–Jones distributions. If random variables of the von Mises distributions in (A) are transformed by equation (10′) shown in (B), the Kato–Jones distributions in (C) are obtained. In (C), the bold/thin lines are used when the transformation displayed by the bold/thin line in (B) was used, and the red/blue line was used when the von Mises distribution displayed by the red/blue line in (A) was used. The transformations in the bottom row are omitted.

Depending on the combinations of the parameters (μ, κ, *r*, ν), the Kato–Jones distribution depicts various shapes (Figure 2). Because the original 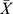
s were concentrated around μ, the 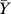
s are shifted to μ+ν; however, the shift is not perfect and the distribution has a mode between μ and μ+ν and a tail toward μ (Figure 2C). If ν is very close to π, *f_KJ_*(*y*; μ, κ, *r*, ν) may be bimodal (the bold line in the middle row), and some combinations of the parameters produce abnormal shapes (the bottom row in Figure 2C and Figure 7D). If ν = 0, transformation (10) just increases the concentration at μ (the middle row in Figure 2C). If ν = *r* = 0, *f_KJ_*(*y*; μ, κ, 0, 0) = *f_VM_*(*y*; μ, κ).

### Heading Model Allowing Asymmetric Distributions

On the basis of the method of generating the distribution, we extended model (4) using the Kato–Jones distribution as follows. The animal originally adjusts the heading directions toward 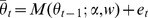 (*e_t_* ∼ VM(0, κ), i.i.d.), or equivalently, toward 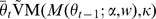 (i.i.d.). External forces may alter the direction of heading. For a flying bird, wind can be a significant force, and 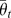 is driven to λ if the wind is blowing to λ. Then, *M*(θ*_t_*_−1_; α, *w*) corresponds to μ, and λ to μ+ν. Thus, if we set 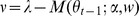 and define.
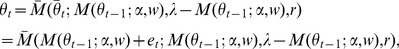
(11)then, θ*_t_* follows the Kato–Jones distribution and can be written as



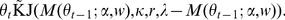
(12)When we have no information on the focal direction of the animal, we set α as an unknown parameter, and the maximum likelihood estimates (MLEs) of the five parameters, α, *w*, κ, *r*, and λ, can be obtained by numerically maximizing the logarithm of the likelihood (conditional on Θ_0_) given by
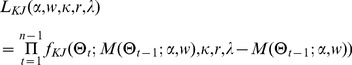
(13)


If the symmetric von Mises distribution or wrapped Cauchy distribution is used, the right-hand side reduces to 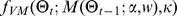 or 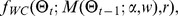 respectively.

Kato and Jones [11] have also investigated the circular–circular regression model in the form of equation (6) assuming *e* ∼ KJ(μ, κ, *r*, ν) (i.i.d.). On the other hand, model (12) is not written in the general form of a regression (*y* = *f*(*x*)+*e*). Instead, *e_t_* in equation (11) is distributed as VM(0, κ) (i.i.d.), and parameter 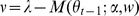 in equation (12) varies with time. Thus, the probability density function of θ*_t_*, 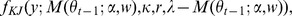 varies depending on the value of θ*_t_*_–1_. We can see changes in these distributions if the graph is drawn for several different θ*_t_*_–1_s. If some of them exhibit abnormal shapes such as bimodal, interpreting them as effects of wind may not be feasible, and we need to consider other interpretations, which are demonstrated in Example 3.

### Examples of Simulated Trajectories

Figure 1C shows examples of trajectories simulated by equation (3) using the heading model (4). The speed was fixed at 1 and focal direction was east (α = 0).

If *w* = 1, equation (4) reduces to the CRW (equation (1)). If 0< *w* <1, an animal adjusts its direction of heading toward α. If *w* is close to 0 a stronger adjustment operates (red −+− in Figure 1C), whereas if *w* is close to 1, several time steps are needed until the animal turns to α (red −Δ− in Figure 1C).

If κ is small, stochastic errors become large and an animal may not adequately adjust its direction of heading, resulting in a wandering trajectory (blue −+− in Figure 1C).

If *w* <0, this corresponds to a linear regression with a negative correlation (−▪− in Figure 1A). θ*_t_* tends to be opposite from θ*_t_*_–1_ with respect to α. Consequently, an animal adjusts its direction of heading reciprocally around α, resulting in a zigzag trajectory (red −▪− in Figure 1C).

Figure 1D illustrates a trajectory simulated by equation (3) using the heading model (12). The focal direction is east–southeast (α = –π/8), however, the animal is forced to move toward north–northwest (λ = 5π/8). The resulting trajectory is going to east (Figure 1D) and visually similar to the trajectory simulated by the heading model (4) (red −+− in Figure 1C). Thus, by just looking at trajectories, we can hardly identify whether the trajectory was produced from a symmetric or asymmetric distribution. These simple examples indicate the need for appropriate statistical analyses, which are demonstrated in Examples 1–3.

Ecological interpretations of the parameters (α, *w*, κ, or *r*) are discussed after all the statistical methods are described. Differences between CRW and C-AR, together with contrasts between the von Mises distribution and wrapped Cauchy distributions, are illustratively described in Appendix S2.

### Speed Model

In general, birds fly faster in the presence of a tailwind and more slowly in the presence of a headwind. For the real trajectory data of the seabird described below, our exploratory analyses revealed that observed speeds (*V_t_*) were strongly correlated with directions of heading (Θ*_t_*), presumably as fast speeds when flying leeward and slow speeds when flying windward. In addition, *V_t_*s were weakly or not correlated with the previous speeds (*V_t_*_–1_) and angular velocities (Θ*_t_* – Θ*_t_*_–1_) depending on the trajectories (examples of these analyses are shown in Appendix S3). Therefore, for a speed model, we applied the following multivariate auto-regressive model:
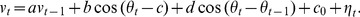
(14)


Here, *a*_,_ *b*, *c*, *d*, and *c*_0_ are unknown parameters (*b* >0, –π ≤ *c*<π), and η*_t_* ∼ *N*(0, σ^2^) (i.i.d.), where *N*(μ, σ^2^) indicates the normal distribution of mean μ and variance σ^2^. The MLEs of the six parameters, (*a*_,_ *b*, *c*, *d*, *c*_0_, σ^2^), can be obtained by the maximum likelihood method (conditional on Φ_0_ and *V*_0_) using the observed directions {Φ*_t_*} and speeds {*V_t_*}.

If speeds are simulated by equation (14), *v_t_* may take a negative value. In order to avoid this problem, *v_t_* must be chosen from the truncated normal distribution (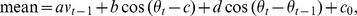 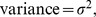 only positive values are taken), and equation (14) should be reformulated using the truncated distribution. However, in the cases of the seabird’s flight trajectories analyzed in this study, MLEs were almost equal, suggesting that equation (14) very closely approximated to the model using the truncated distribution. This was because the seabird maintained high speeds during flights, and in fact, when we simulated speeds, negative values were produced with very low frequencies.

Therefore, for simplicity, this study used equation (14) as an approximation in the maximum likelihood method, and when speeds were simulated, we continued simulation until a positive value was obtained (this was achieved mostly at the first attempt, and in almost all cases within two attempts).

### Ethics Statement

Field studies were conducted under permission from the Ministry of the Environment and the Agency for Cultural Affairs, government of Japan, and the Ethics Committee of the University of Tokyo.

### Study Site and GPS Data on a Seabird

The models described were applied to GPS location data for a breeding seabird (streaked shearwater: *Calonectris leucomelas*) on Sangan Island, Japan (39°18′N, 141°58′E). Streaked shearwaters in this area have been previously studied by Yamamoto et al. [14], [15] and Shiomi et al. [16].

In this study, a bird was captured at nighttime on September 21, 2007 (male, 590 g). A GPS logger (Technosmart, Italy, 28 g) was deployed along the median line of the back and the bird was released. It was recaptured after it returned from a 1-day foraging trip on the Pacific Ocean [16]. GPS coordinates were recorded every 0.5 s from September 21 at 22∶39 to September 22 at 22∶35.

Because GPS coordinates were measured continuously at frequent intervals, the trajectory we obtained was almost smoothly connected [3]. For convenience, we transformed longitude–latitude records into *x-y* coordinates on the meter scale (the UTM coordinate system) with Igor Pro (ver. 5.0, WaveMetrics, Inc.) and Ethographer [17]. The GPS data are freely available upon request.

**Figure 3:**
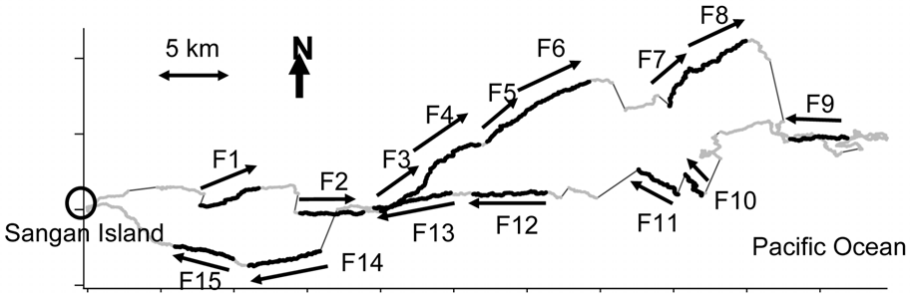
GPS trajectory of an adult *Calonectris leucomelas* breeding on Sangan Island. The bold lines represent the 15 selected flight sections (F1–F15), while the gray lines represent other flying trajectories. The arrows indicate the entire observed flight direction 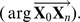 GPS coordinates when the bird appeared to be on the sea surface are not displayed, and the two flights are connected by the thin line.

To demonstrate the proposed models, we arbitrarily selected visually oriented and homogeneous segments of 500 s (1001 locations) from periods when the bird was considered to be airborne and continuously flying (observed speeds were mostly >4.0 m/s, cf. [16]). The selected 15 flight sections were labeled as F1–F15 (Figure 3) and categorized as follows: morning flights near the island, presumably traveling to foraging areas (hereafter “outward flights,” F1–6), daytime flights on the Pacific Ocean, presumably searching for food (hereafter “searching flights,” F7–11) and afternoon to evening flights returning to the island (hereafter “homeward flights,” F12–15) (Figure 3).

One of the most elementary summary statistics for movement trajectories is the total flight length 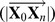 (shown in the third row in Appendix S4). F2 and F15 had similar distances (4322.6 m and 4069.1 m, respectively); however, considerable differences actually existed and identifying such characteristics is one of the most elementary tasks for applying the model (Examples 1 and 2). F10 clearly had a shorter flight distance and was weakly oriented but was highly tortuous. The reason for selecting F10 was to demonstrate the applicability of the model to such abnormal patterns.

### Time Unit

The GPS data were taken at 0.5 s intervals. If we resampled the data as **X**_0_, **X**_2_, **X**_4_, …, **X**_1000_, we can apply the models to data for “time units” of 1 s. If resampled as **X**_0_, **X**_4_, **X**_8_, …, **X**_1000_, the time unit becomes 2 s. Statistically, in the heading model (4), *e_t_* is assumed to be independently and identically distributed; therefore, the residuals Θ*_t_* − *M*(θ*_t_*_–1_) should not be correlated with Θ*_t_*_–1_ − *M*(θ*_t_*_–2_), i.e., auto-correlations should be absent. For model (12), because *e_t_* in equation (11) is independently and identically distributed, 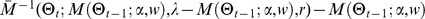 should not be correlated, where
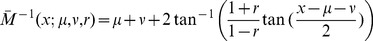
(15)is the inverse transformation of 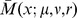
(equation (10′)).

Therefore, we resampled the data of each 500-s flight section for every *T* second (*T* = 1, 2, …, 10) and denoted them as {**X** *_t_*} = (**X**_0_, **X**_1_, …, **X** *n_T_*) (*n*_1_ = 500, *n*_2_ = 250, *n*_3_ = 166, …, **X** *n_T_* = **X**_1000_ if 500 is divisible by *T*). This produced 10 time-series data of length *n_T_* +1. In this study, *T* is referred to as a “time unit.” We calculated MLEs for these 10 data sets, and initially, we visually checked the absence of auto-correlations using scatter diagrams. We then approximated circular residuals by white noise and used the auto-correlation coefficient of time-lag one (denoted by 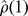); if 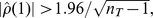 the model was not applicable due to the presence of auto-correlations ([18], section 1.4.1). The ecological meaning of a time unit is discussed later.

### Computation and Model Selection

The maximization of the logarithm of the likelihood equations (13) and the log-likelihood of model (14), conditional on Φ_0_ and *V*_0_, were conducted by the quasi-Newton method using the “Maximize” command in Mathcad (ver. 14, Mathsoft, Inc.). The computation began with several different initial values and we then checked whether the true maximum log-likelihood was obtained with high precision, as was suggested for the Kato–Jones distribution in [11].

Because CRW, C-AR, and the three circular distributions contain different numbers of parameters, the models were evaluated by the Akaike information criterion (AIC) [19], [20]:
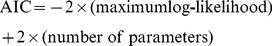
(16)


The model with the smallest AIC was selected. The covariates in model (14) were also selected by the AIC.

### Variation of Models

As previously mentioned, birds tend to fly faster in the presence of a tailwind and more slowly in the presence of a headwind. If the speed model (14) takes the maximum value at θ*_t_* = *c*, *c* may be used as the leeward direction. Thus, we have applied C-AR using the Kato–Jones distribution while fixing 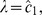 where 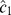 is the MLE of *c* when the time unit is 1 (*T* = 1). If this model shows the smallest AIC value, we may interpret the model as described above. If not, the MLE of λ may not be in the leeward direction, and we need another interpretation, which is demonstrated in Example 3.

When a bird returns to the nest, it has a specific “focal point” rather than a focal direction. In such cases, let **X** *_fp_* be the location of the focal point. The direction from the current location **X** *_t_* to the focal point is given by 
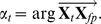
 Then, we can modify the model by changing *M*(θ*_t–_*_1_; α, *w*) to
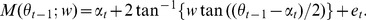
(17)


We applied this model to the homeward flights, for which **X** *_fp_* = {the location of the island}.

If a bird intended to fly toward α and succeeded with its navigation, the resulting flight direction, 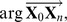 was close to α. Thus, if the bird was actually flying as it navigated, the MLE of α should be close to 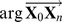. Hence, we have applied the model by fixing 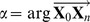. If this model has a smaller AIC value, we may interpret that the bird was flying as it navigated.

### Notation

We denoted CRW using the von Mises distribution by CRW(VM), C-AR using the Kato–Jones distribution by C-AR(KJ), and so on. The MLE of a parameter was denoted by using ^∧^, and we added the flight number or/and time unit when needed (e.g., 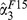). A model having some constraint was denoted by 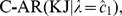 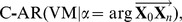 and so on.

### Goodness-of-Fit for Global Patterns

The maximum likelihood methods for equations (13) and (14) focused on fitting for two consecutive time steps. Whether the selected model can reproduce global patterns similar to the observations is uncertain. This study introduced the following two goodness-of-fit tests.

Heading distribution: whether the distribution of directions of heading {θ*_t_*} simulated by the model had a distribution similar to the observed heading distribution {Φ*_t_*}.

Final locations: whether the model can predict the observed final location, i.e., the simulated final points 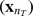 are sufficiently close to **X** *n_T_*.

For the first goodness-of-fit, beginning with the observed first direction of heading 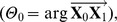 we simulated a circular series {θ*_t_*} (*t* = 1, 2, …, *n_T_*) 1000 times. Here, random circular variables from the von Mises and wrapped Cauchy distributions were produced by a rejection method. A random variable *x* from the uniform distribution –π ≤ *x*<π was rejected if 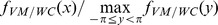 was smaller than a randomly chosen number from 0 to 1 or was accepted if it was greater. Random variables of the Kato–Jones distribution were produced by transforming random variables of the von Mises distribution by equation (10).

The interval –π ≤ θ<π was divided into 24 classes. Let *S_d,k_* be the number of θ*_t_*s in the *d*-th class for the *k*-th simulated series (*d* = 1, 2, …, 24, *k* = 1, 2, …, 1000). Let *E_d_* be the average over {*S_d,k_*} and *O_d_* the observed number of Θ*_t_*s in the *d*-th class. We calculated Σ(*E_d_* – *O_d_*)^2^/*E_d_* and conducted a χ^2^-test to obtain the *P*-value (classes with *E_d_* <3 were excluded). We also showed *O_d_*s, *E_d_*s, and the 95% confidence envelopes by connecting the 2.5 and 97.5 percentiles of {*S_d,k_*} for each *d*.

For the second goodness-of-fit, using the simulated {θ*_t_*} and the observed first speed 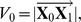 we simulated a speed series {*v_t_*} using equation (14) (only positive values were used). Starting at **X**_0_, we then drew 1000 movement trajectories {**x** *_t_^k^*} (*t* = 1, 2, …, *n_T_*). Let **M** = (*M*_1_, *M*_2_) be the mean for the final locations 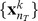 and **∑** = variance–covariance matrix for 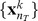 (*k* = 1, 2, …, 1000). By assuming that the final locations 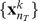 were approximately distributed in a two-dimensional normal distribution with mean **M** and variance–covariance matrix ***∑*** (let denote this probability density function by *f_N_*(**y**)), we calculated the probability of obtaining the observed final location (**X** *n_T_*) by
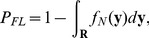
(18)where **R** refers to the region satisfying *f_N_*(**y**) ≥ *f_N_*(**X** *n_T_*) (inner part of the ellipse centered at **M** and passing **X** *n_T_*). If *P_FL_* <0.05, the model was rejected.

### Distribution of Maximum Likelihood Estimates

MLEs have asymptotic normality, and we estimated their distribution from the Fisher information matrix, which was useful when we compared estimated parameter values and looked for significant differences between trajectories.

The Fisher information matrix was not derived for the Kato–Jones distribution (a specific case was shown in [11]). In addition, a sufficient sample size for the asymptotic normality is unknown. Thus, we conducted simulation-based inferences. For simulated trajectories produced by the selected model, we conducted the maximum likelihood method again, obtained MLEs for each simulated trajectory, calculated standard deviations over these estimates, plotted their distributions, and checked if the estimates were symmetrically distributed with a center at the true value. This is demonstrated in Example 2.

### Correspondence between the Model and the Conceptual Framework

Nathan et al. [1] summarized the general conceptual framework for movement ecology as consisting of four basic components: internal state, navigation capacity, motion capacity and external factors.

In our modeling framework, focal direction α and final point **X** *_fp_* are directly related to navigation capacity, and the constraint 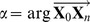 examines the bird’s execution for navigation. Alternatively, assuming that the bird has sufficient navigation capacity, we may interpret that the animal established a “dummy” direction α so that the wind would push its flight course toward the true objective direction.

When C-AR(KJ) is applied to a flying bird, λ estimates the leeward direction and *r* quantifies wind effects, which contribute to the quantitative evaluation about the external factors. The constraint 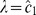 ascertains if the main external factor was wind.

As demonstrated in Figure 1, *w* quantifies a bird’s intention to fly toward the focal direction α (navigation intention), but when the time unit *T* is short, *w* is also influenced by a physical limitation in turning (motion capacity). Similarly, κ quantifies the effort the bird expends in accurately adjusting the direction of heading (navigation intention), or the capability to accurately control the direction of heading (motion capacity). Hence, (*w*, κ) is related to the internal state, motion capacity, and navigation capacity, and as far as only trajectory data are available, separate quantification seems to be difficult.

The time unit is related to motion capacity. The presence of auto-correlations in residuals means that physical and physiological constraints frequently prevented a bird from controlling the direction of heading, and that the time-scale is finer than the motion capacity. If no auto-correlation exists in the residuals, the animal might control the flight at that average pace.

Our modeling thus aims to integrate the general conceptual framework for oriented movements at short time-scales into statistical modeling and provides its (partial) quantification.

## Applications to GPS Data

For time units shorter than 3 s (*T* <3), all 15 flight sections had either an insufficient goodness-of-fit or the presence of auto-correlations in the residuals. For *T* = 3, we found a satisfactory model in 11 flight sections, and all the 15 flight sections had a satisfactory model for *T* ≤6. The MLEs for the smallest time units at which satisfactory models were found are summarized in Appendix S4.

In the following sections, we use five examples from the three flight types to explain the basic properties of the proposed models (primarily the heading model) and demonstrate how to characterize a given trajectory by the MLEs and how to interpret them ecologically.

### Example 1: Outward Flight

**Figure 4:**
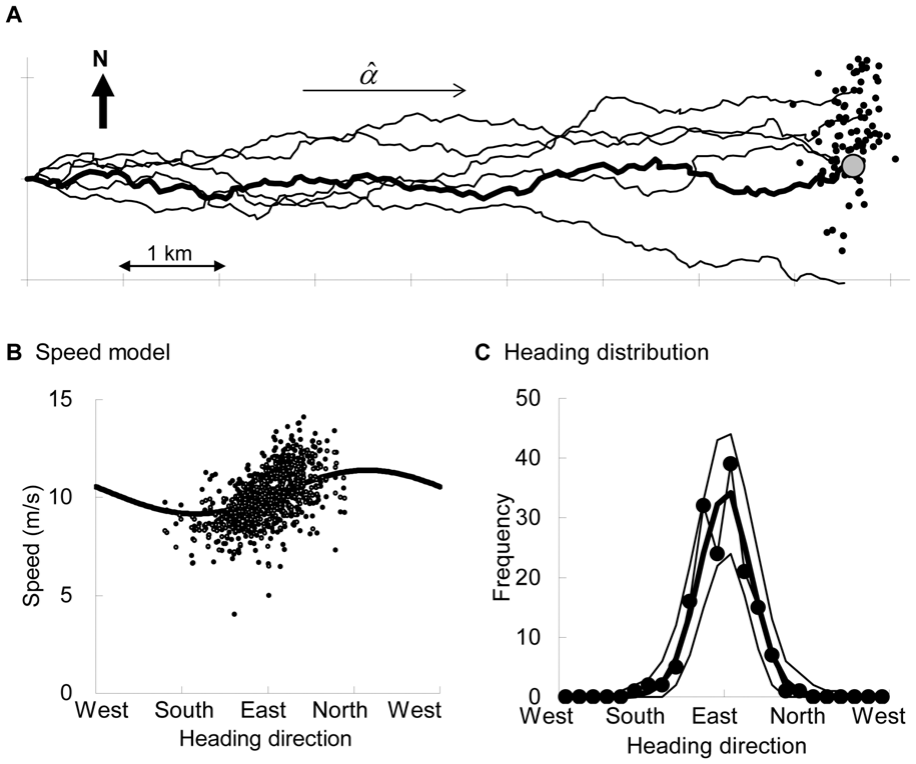
Example 1: outward flight. (A) Observed GPS trajectory of F2 (bold line, the final location is indicated by the large gray circle), five examples of simulated trajectories (thin lines), 100 examples of the final locations (dots) of 1000 simulated trajectories produced by the selected model. (B) Black dots: Points from the scatter diagram showing the relationship between speed and direction of heading. White dots: Predicted speeds by the selected model from (Θ*_t_*, *V_t_*_–1_, cos(Θ*_t_* – Θ*_t_*_–1_)) plotted on Θ*_t_*. Bold line: The expected speed as a function of the direction of heading when the previous speed was fixed as the mean over the flight section and angular velocity was 0. (C) The heading distribution: Observed trajectory (─•─), and mean (bold line) and 95% confidence envelopes (thin lines) derived from 1000 simulated trajectories.

For F2 (the bold line in Figure 4A), Figure 4B illustrates the scatter diagram between observed directions of heading (Θ*_t_*) and speeds (*V_t_*) and predicted speeds of the selected speed model for *T* = 1. From this speed model, we obtained 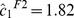 and assumed this direction as leeward.

For *T* = 2–3, AIC selected the heading model of 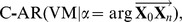 and auto-correlations in the residuals were present for *T* = 2 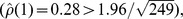 whereas they disappeared for *T* = 3 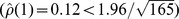 (the scatter diagrams are shown in Appendix S5). Combined with the selected speed model at *T* = 3 (this is shown in Appendix S6), the simulated trajectories showed oriented patterns similar to the observed pattern. The observed final locations were distributed around the center of the simulated final locations (Figure 4A, *P_FL_* >0.7), and the heading distribution also showed a good fit (*P*>0.4, Figure 4C).

Therefore, we interpreted that the bird was flying eastward and approaching the searching area, and at least 3 s were required to control the direction of heading.

The same model type, 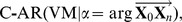 was selected for the subsequent outward flight section (F3, Appendix S4).

### Example 2: Homeward Flights

**Figure 5:**
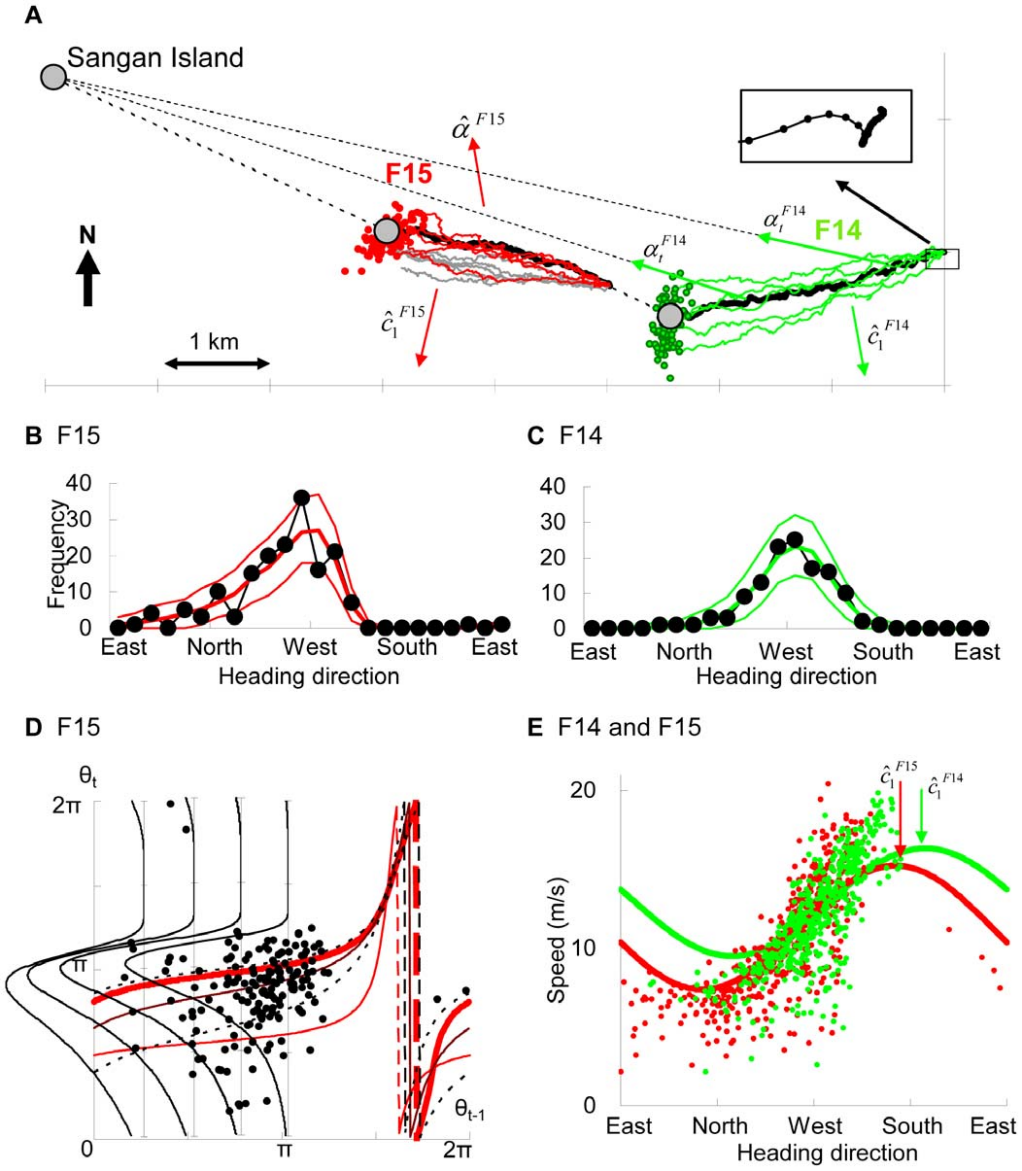
Example 2: homeward flights. (A) GPS trajectories of F14 and F15 (bold lines), 5 examples of simulated trajectories of each selected model (thin lines), and 100 examples of final locations of 1000 simulated trajectories produced by each selected model (green: F14, red: F15, throughout). The gray lines indicate 5 examples produced from the model that were not selected for F15 (setting the island as the focal point). The inset enlarges the takeoff section. (B, C) Heading distribution; the observed heading distribution (─•─), and mean (bold line) and 95% confidence envelopes (thin lines). (D) The red thin, red bold, thin, and dotted lines represent the regression curves, modes, medians, and 25% and 75% quartiles of the selected model for F15, respectively. Dots are points from the scatter diagram indicating the direction of heading (Θ*_t_*_–1_, Θ*_t_*). The four rotated curves indicate the density functions of the Kato–Jones distributions for θ*_t_* when θ*_t_*_–1_ is π/4, π/2, 3π/4, and π. (E) A scatter diagram showing the relationship between the direction of heading and observed speed (*T* = 1) and the expected speed when *V_t_*_–1_ = {the mean over each flight section} and Θ*_t_* – Θ*_t_*_−1_ = 0.

The second examples are F14 and F15. These are parts of the same final flight (Figure 3). From human visual examination, the trajectories of F2, F14, and F15 in Figure 3 and the bold lines in Figures 4A and 5A appeared similar; however, they had significantly different properties.

For these flight sections, speeds were essentially determined by the direction of heading, and previous speeds and angular velocities played a relatively small role (Figure 5E). The selected speed model provided 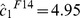 and 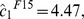 suggesting that the wind direction was from the northeast to southwest. The inset in Figure 5A enlarges the takeoff section of the flight. In general, when taking off, a seabird is running in a windward direction, and the start toward the north supported the estimation of the above leeward direction.

For F14, 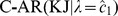 setting the island as the focal point was selected, and no significant auto-correlation 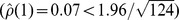 was observed at *T* = 4. On the other hand, for F15, trajectories produced by this model type did not approach the island but were biased to the estimated leeward direction (gray lines in Figure 5A). The selected model was 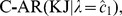 and auto-correlations disappeared at *T* = 3 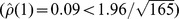 (Appendix S5). The observed heading distributions were skewed (─•─ in Figures 5B and C), but this was weaker for F14, and the heading distributions produced a sufficient fit (*P*>0.8 for F14, *P*>0,1 for F15). Combined with the selected speed models (Appendix S6), the final locations displayed sufficient goodness-of-fit for both flights (Figure 5A) (*P_FL_* >0.8 for both).

Figure 5D illustrates the scatter diagram between the observed heading directions Θ*_t_*_–1_ and Θ*_t_* for F15, together with the regression curve, modes, medians, and 25% and 75% quartiles of the selected 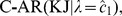 and examples of the probability density functions of the Kato–Jones distributions for θ*_t_*. Because Kato and Jones [11] did not analytically derive the mode of *f_KJ_*(*y*), we numerically computed the modes, while the medians and quartiles were calculated by transforming those of 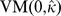
using equation (10). These quantitatively show the asymmetry in the direction of heading.

**Figure 6:**
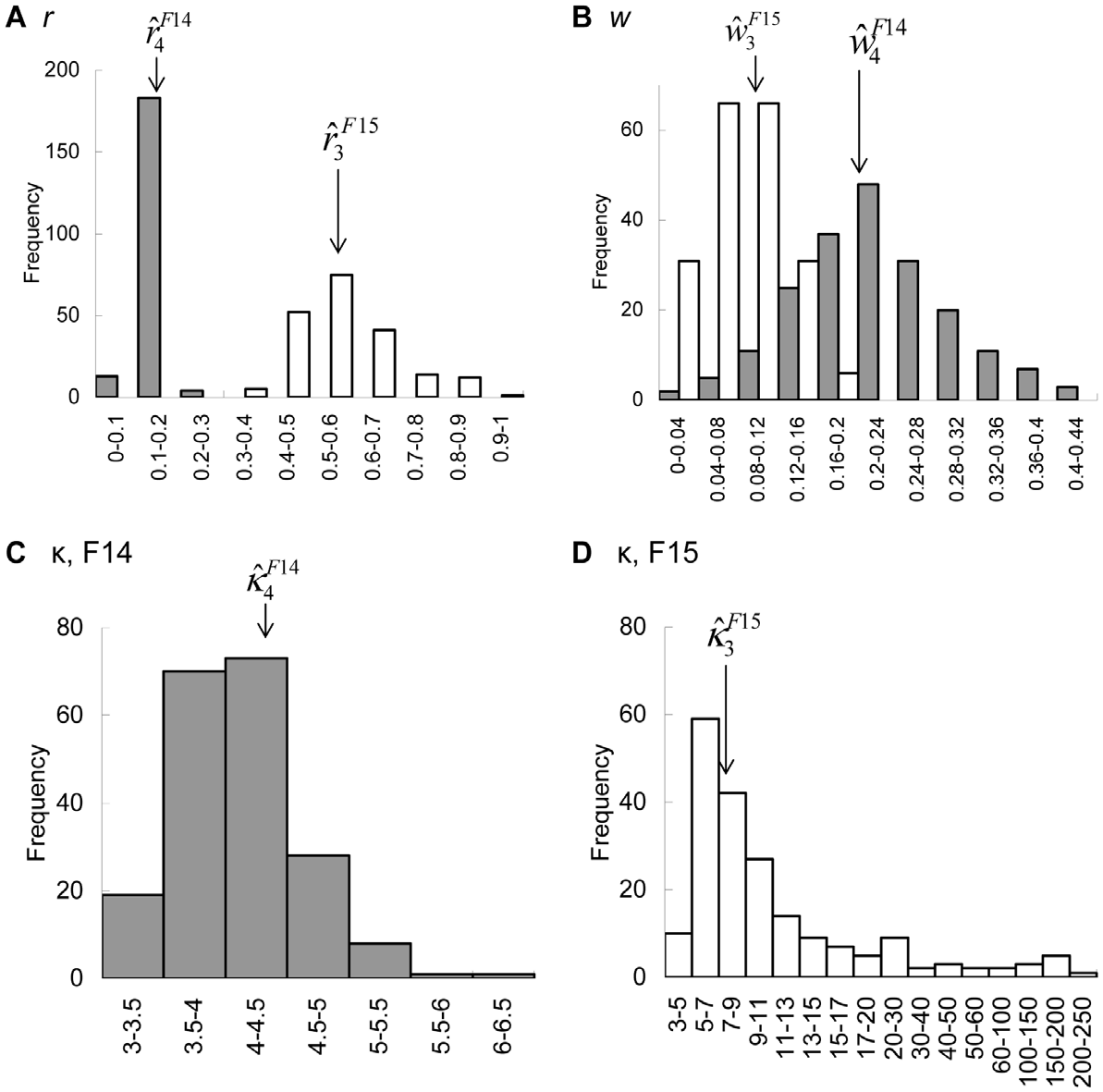
Distributions of the maximum likelihood estimates. The horizontal axes indicate the classes of each parameter, and the vertical axes show the frequencies from 200 simulations. Shaded: F14, white: F15. The arrows indicate the true parameter values.

The distributions of the MLEs obtained from 200 simulated trajectories using the MLEs of each selected model are shown in Figure 6. The distributions of 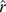
s were close to a normal distribution and were clearly separated between the two models (the standard deviation (SD) was 0.028 for 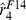 and 0.12 for 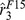, Figure 6A), whereas the distributions of 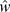 overlapped to some degree (SD = 0.076 for 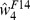 and = 0.039 for 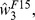 Figure 6B). The distribution of 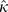 for F14 was also close to a normal distribution (SD = 0.50), whereas the distribution for F15 had a fat tail for large values (SD = 33.2). The right-skewed fat tail is likely to occur because a small change in κ values does not influence the von Mises distribution when κ is large.

Based on the selected models and their MLEs, we contrastively interpreted the two flights as follows. During F14, the bird was likely to have less intention to fly toward the island (greater 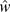). The bird was flying in directions whereby it could control the flight easily and was likely to put less effort into navigation (smaller 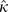). As a result, the trajectory was moved to the south by the wind and the bird failed to approach the island. The bird recognized this miss-navigation and established a “dummy” focal direction toward the north 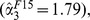 more easterly than the objective island, and was flying under stronger wind conditions during F15 (greater 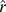). The seabird might increase the pace for controlling its direction of heading from every 4 s on average to every 3 s, although these time units were the minimum intervals for the acceptable models and may not coincide with the true pacing. Even though, the MLEs and their distributions for *T* = 4 were close to those for *T* = 3 (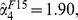 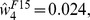 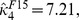 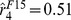), thus, the same argument as above is valid.

For the other two homing flights, F12 and F13, the model setting the island as the focal point was not selected, either, and the same model as F15 was selected (Appendix S4).

### Example 3: Searching Flights

**Figure 7:**
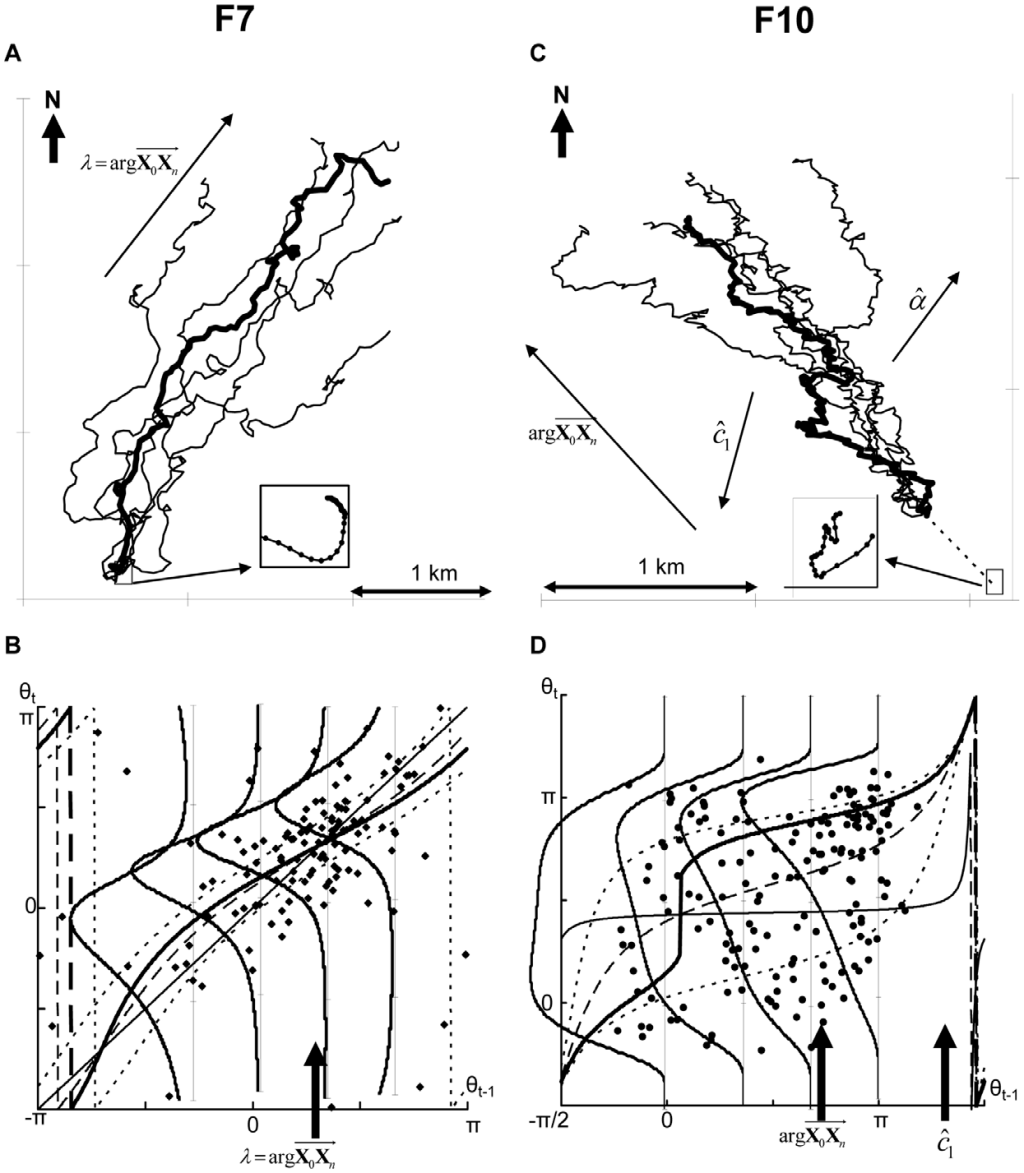
Example 3: searching flights. (A, C) Observed GPS trajectory of F7 (A) and F10 (C). The bold lines are observed trajectories and the thin lines are five examples of simulated trajectories for each selected model. The insets enlarge each takeoff section. (B, D) The thin, bold, dashed, and dotted lines represent the regression curves, modes, medians, and 25% and 75% quartiles of each selected model, respectively. The dots represent points from scatter diagrams between Θ*_t_*_–1_ and Θ*_t_* for every 4 s (B) and 3 s (D). The four rotated curves are the density functions of the Kato–Jones distributions for θ*_t_* when θ*_t_*_–1_ is −π/3, 0, π/3, and 2π/3 (B) and 0, π/3, 2π/3, and π (D).

For F7, very weak correlations existed between the speeds and directions of heading (Appendix S6). In addition, the bird was taking off to the south without changing the direction (before taking off, the seabird had been floating on the sea surface from the north to the south, the inset in Figure 7A). Hence, presumably little wind occurred during this flight.

For time unit *T* = 4, 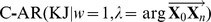 was selected and no significant auto-correlations were observed in the residuals. Although C-AR setting *w* = 1 is equivalent to CRW, the actual trajectory is oriented (Figure 7A), and although the Kato–Jones distribution was used, the observed Θ*_t_*s were symmetrically distributed with respect to the modes and the graphs of
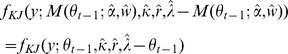
also appeared to be symmetric (Figure 7B). This strange model selection was a result of unequal “variances” (a variance as in a second-order moment is not defined for circular variables, but here for simplicity, we used the term “variance” to mean the degree of concentration/diversification). When the bird was flying to 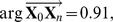 subsequent directions were concentrated around 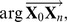 while Θ*_t_*s were diverse when Θ*_t_*_–1_ was distant from 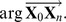 These unequal variances prevented C-AR(VM) and C-AR(WC) (*e_t_* ∼ VM (i.i.d.) or ∼ WC (i.i.d.) means an equal variance) from displaying a sufficient goodness-of-fit, and the Kato–Jones distribution was used to flexibly capture unequal variances.

In such cases, λ and *r* should not be interpreted as wind effects, but they do have the same role as α and *w*: λ was the focal direction, and *r* quantified the intention of the bird. Therefore, 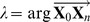 indicates that the bird was flying as it navigated.

Based on these characterizations of the trajectory, we hypothesized that the bird flew in a northeast direction for searching and occasionally flew in abnormal directions.

The same model type, 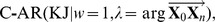 was selected for three outward flight sections (F4, F5, F6, Appendix S4), and a diagram similar to Figure 7B indicated that the Kato–Jones distribution could be used for the same purpose, i.e., for capturing unequal variances.

Another searching flight, F10, appeared to be highly tortuous and contained a looping pattern (Figure 7C). Speeds were strongly correlated with the direction of heading (Appendix S6). The observed directions of heading were diverse, but southwest to southeast directions were avoided (Figure 7D and Appendix S6). 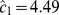 was obtained from the speed model at *T* = 1, which was close to the takeoff direction (inset in Figure 7C). For the heading model at *T* = 3, 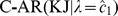 was selected. The estimated focal direction 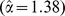 was more eastward than the entire observed flight direction 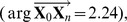 and a large 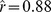 suggested strong wind effects. 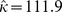 was extremely large and produced abnormal shapes, as seen in the four graphs in Figure 7D. The degree of concentrations decreased when θ*_t_*_–1_ was distant from 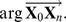


We made an interpretation of this flight as follows: The seabird flew to the northwest while searching in an area where a strong wind was blowing. The searching flight covered diverse directions but leeward directions were avoided.

## Discussion

### Circular Statistics and Movement Ecology

Applying the recently proposed circular auto-regression and the recently proposed Kato–Jones distribution, we proposed a flexible movement model that can explain diverse oriented trajectories and quantitatively characterize each pattern under the conceptual framework of movement ecology. Each trajectory exhibited specific characteristics, and the statistical models considerably helped us understand the movements. The above examples also suggest that the actual movement patterns of animals, even those that are just visually oriented, are much more diverse than we predicted.

In particular, the heading model (12) enabled us to evaluate the seabird’s internal states such as navigation capacity separately from external factors such as winds. Movement ecology will advance in parallel to developments in circular statistics, and the development of circular statistics will be promoted by the practical demands made from movement ecology.

### Future Developments

The role of the Kato–Jones distribution is not limited to separate external effects. It can also be used for flexibly capturing abnormal distributions (Figure 7D) and unequal variances (Figure 7B). For the latter, an alternative is to formulate κ by some function [21], and this approach can be extended to include other covariates such as environmental conditions and landscape information [1], [5], [21], [22]. The heading models used in the general discrete-time modeling framework in [22] are basically equivalent to equation (2) in this paper. Therefore, the same general framework can be expected to be applicable if the circular auto-regression is used as a heading model.

The proposed movement model is particularly useful if the direction of heading plays a central role in the animal’s movement (e.g., a flying bird whose speed was strongly correlated to the direction of heading). If speeds also play a crucial role (e.g., walking/running animals such as horses), or if a long-term trajectory was taken that contained times when an animal stopped moving, a more sophisticated speed model than (14) is needed [21], [22].

For the purpose of demonstration, this paper artificially selected seemingly homogeneous oriented periods of movement. The applicability of a heading model (12) is not limited to oriented trajectories. For example, gradual curves to a focal direction and an area-restricted search around a focal point with looping can also be realized (Appendix S7). Therefore, the next step is to establish statistical methods that adequately divide a trajectory into segments, referred to as short-duration fundamental movement elements in Getz and Saltz [2], so that each segment can be explained by a single model [23]. Another important development would occur if the proposed models are applied at different time-scales [1], [9], [24]. This paper focused on fine time-scales. If similar analyses are applied to longer scales (e.g., minutes, hours, or days) the parameters would have other ecological interpretations.

Time unit selection is another issue related to time-scales. In this study, we found that a streaked shearwater required an average of 3–4 s to control its direction of heading. However, this was a minimum time-scale regarding motion capacity and the true time-scale could be longer. Currently, we have not established a statistical theory for selecting the exact time unit at which a bird did control its flight.

### Null Model for Movement Ecology

The proposed models will work not only for quantifying given movement patterns under the conceptual framework, but also as a null model [8], [25]. Most previous studies used CRW as a null model, and the significant deviation from CRW implied the presence of some specific behaviors, although such deviations have been so frequently observed [6], [7], [9]. If a null model covers most known movement patterns, significant deviations suggest the presence of some unknown patterns. The proposed model covered many known animal movement patterns from correlated random walks, oriented movements, tortuous trajectories, and area-restricted searches around a focal point. Deviations from any of these will explore animal behavior that is currently not well understood. Furthermore, our approach was made on the basis of the general conceptual [1] (and modeling [22]) framework for movement ecology. We therefore expect that our method will be applicable as a basic model in movement ecology.

## Supporting Information

Appendix S1 **Example of an oriented movement model and problems with its application.**
(PDF)

Appendix S2 **Differences between correlated random walk and circular auto-regression and contrasts between the von Mises and the wrapped Cauchy distributions.**
(PDF)

Appendix S3 **Correlations between speeds and the covariates.**
(PDF)

Appendix S4 **Maximum likelihood estimates of the selected models for the 15 flight sections.**
(PDF)

Appendix S5 **Presence/absence of auto-correlations in residuals.**
(PDF)

Appendix S6 **Correlations between the direction of heading and speed and predictions of the selected speed models.**
(PDF)

Appendix S7 **Two examples of non-oriented trajectories produced by circular auto-regression.**
(PDF)
