# Supplementary material for: Toward the Quantification of a Conceptual Framework for Movement Ecology Using Circular Statistical Modeling
Source: PLoS One. 2012 Nov 30;7(11):e50309. doi: 10.1371/journal.pone.0050309 (PMC3511459; doi:10.1371/journal.pone.0050309)
Supplement: Appendix S1 — Example of an oriented movement model and problems with its application. (PDF) [file pone.0050309.s001.pdf]

Appendix-S1. Example of an oriented movement model and its problems

Here we consider the movement model defined by equation (2);

$\theta_t = (1 - w)(\alpha - \theta_{t-1}) + \theta_{t-1} + e_t,$ (2)

in which θ_t is the heading direction from time t to $t + 1$, α is the focal direction, and w is an unknown parameter.

When θ_{t-1} is close to α, this model seems reasonable (Fig. S1A). However, if θ_{t-1} is near $\alpha + \pi \equiv \alpha - \pi \pmod{2\pi}$, equation (2) causes ecologically unreasonable movements; very similar previous directions, ψ_{t-1} and φ_{t-1}, result in almost opposite directions in the subsequent step (B). In other words, equation (2) is not continuous at $\alpha + \pi$, and the discontinuity becomes clear if equation (2) is graphically displayed as (C). The discontinuity makes the model mathematically not so tractable.

Kato's circular auto-regressive model resolves this discontinuity by using the sigmoidal curve shown in (C) ($w = 0.5$, $\alpha = \pi/3$ in equation (4)).

Note: More precisely, θ_t is given by equation (4) if $\theta_{t-1} \neq \alpha + \pi$, and $\theta_t = \alpha + \pi + e_t$ if $\theta_{t-1} = \alpha + \pi$. This inconvenience is resolved if the equation is expressed by complex numbers; then, equation (5) is known as the Möbius transformation [S1].

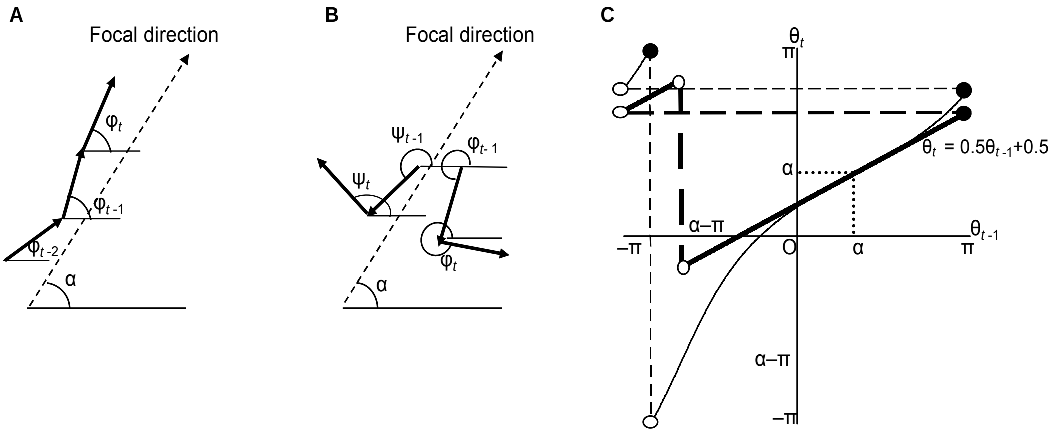

Figure S1. Illustrative explanation on model (2) and its problems. In (C), α = π/3, w = 0.5. ○···○ is used for showing discontinuity, while continuously connected parts as circular variables are connected by ● - - ○.

References

S1. Kato S, Shimizu K, Shieh GS (2008) A circular-circular regression model. Stat Sini 18: 633-645.
