# Supplementary material for: Toward the Quantification of a Conceptual Framework for Movement Ecology Using Circular Statistical Modeling
Source: PLoS One. 2012 Nov 30;7(11):e50309. doi: 10.1371/journal.pone.0050309 (PMC3511459; doi:10.1371/journal.pone.0050309)
Supplement: Appendix S2 — Differences between correlated random walk and circular auto-regression and contrasts between the von Mises and the wrapped Cauchy distributions. (PDF) [file pone.0050309.s002.pdf]

Appendix-S2. Differences between correlated random walk and circular auto-regression and contrasts between the von Mises and the wrapped Cauchy distributions

In the case of CRW, once, an orientation far from α is accidentally simulated, an animal will move in that direction for a while, and there is no mechanism for re-orienting towards α. Hence, even the simulation began with $\theta_0 = \alpha$ (= 0) and used the strictly concentrated von Mises distribution (red line in (B)), sooner or later, simulated θ_ts become distant from α (red line in Fig. S2C) and the trajectory does not move toward α (red line in (A)), even when. In contrasts, in the case of C-AR (w = 0.2), even if an abnormal direction simulated from the less concentrated von Mises distribution (blue line in (B)), subsequent directions soon come back to α (blue lines in (A), (C)).

In general, the wrapped Cauchy distribution is more strictly concentrated around μ and has a fatter tail than the von Mises. Therefore, if the sharply concentrated distribution (green (B)) is used, the fatter tail occasionally accepts an abnormal direction (blue line in (C)). Consequently, the apparently different probability distributions (blue vs. pink in (b)) produced similarly moving trajectories (blur vs. in (A)).

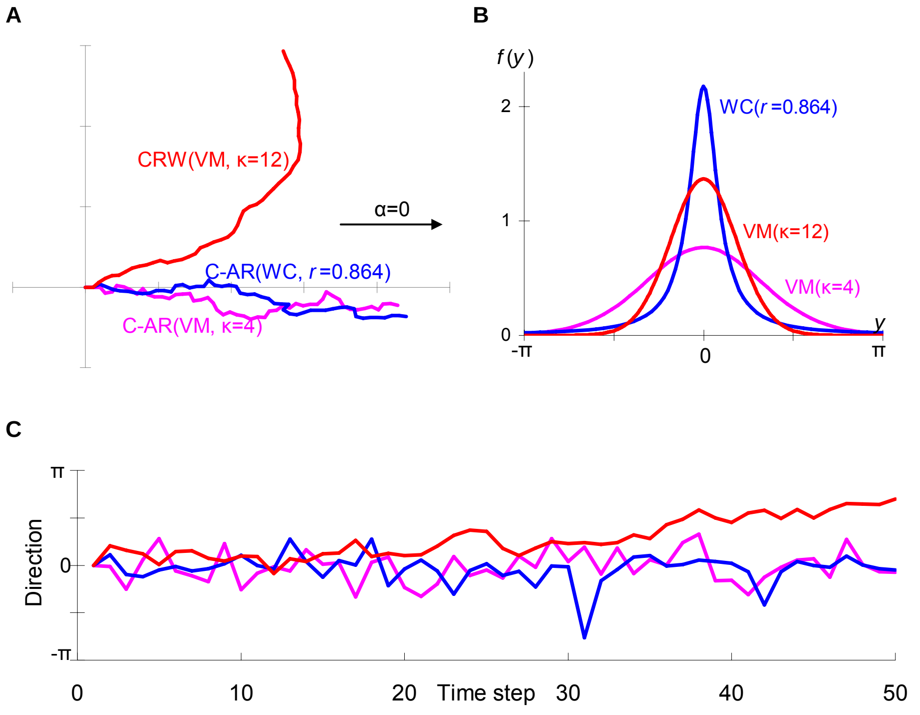

Figure S2. (A) Examples of simulated trajectories. Speeds were fixed at one. The first 50 paths are shown. The grid unit is 10. (B) The probability distributions used in the three models. (C) The changes in simulated heading directions of the three models. The same colors as (A) are used in (B) and (C).
