# Supplementary material for: Toward the Quantification of a Conceptual Framework for Movement Ecology Using Circular Statistical Modeling
Source: PLoS One. 2012 Nov 30;7(11):e50309. doi: 10.1371/journal.pone.0050309 (PMC3511459; doi:10.1371/journal.pone.0050309)
Supplement: Appendix S3 — Correlations between speeds and the covariates. (PDF) [file pone.0050309.s003.pdf]

Appendix-S3. Correlations between speeds and the covariates.

Observed speeds were strongly correlated with heading distributions, weakly or not correlated with previous speeds and angular velocities depending on flight sections and time unit.

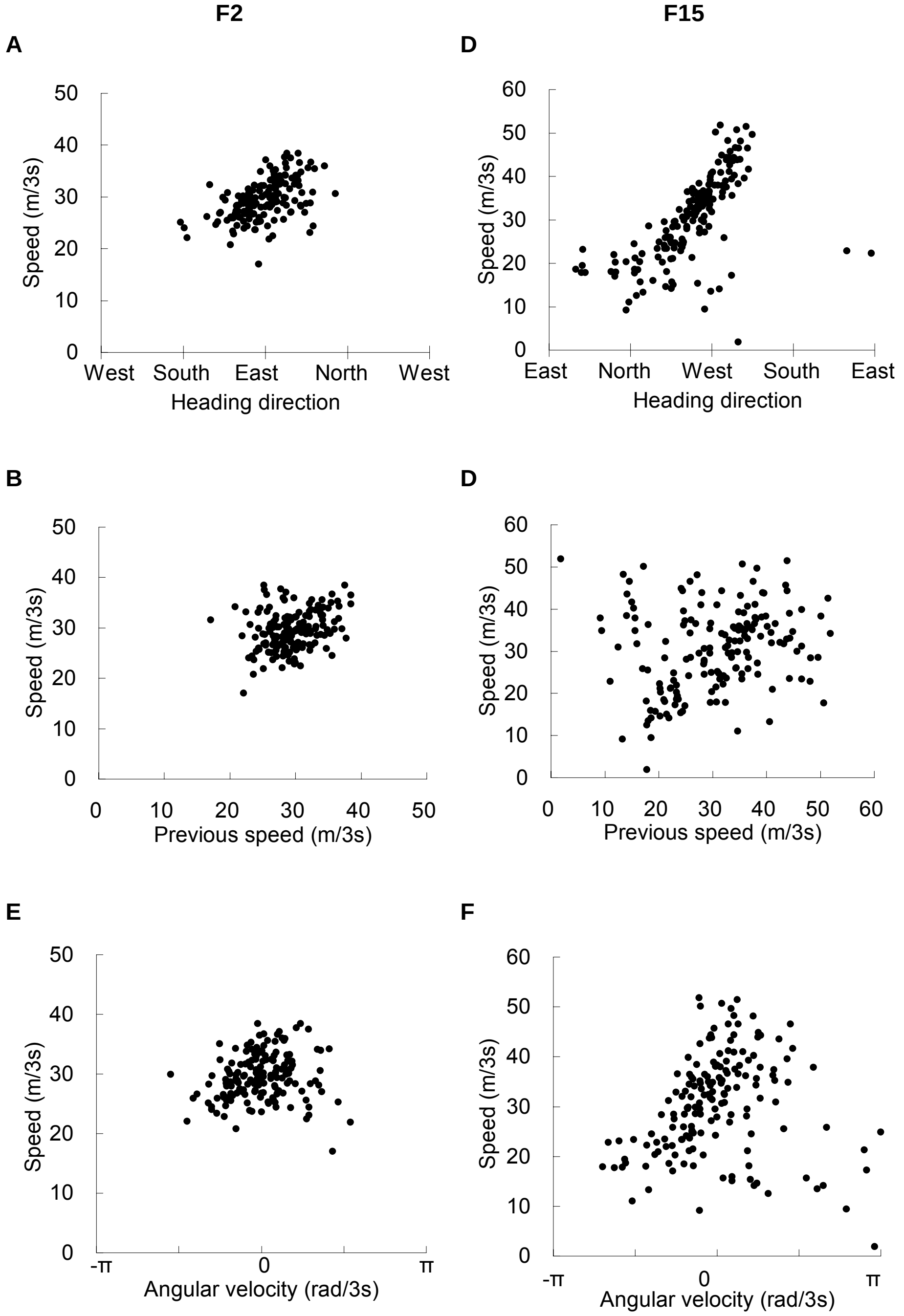

Figure S3. Scattering diagram between speeds (V_t) and heading directions (Θ_t, the top row), previous speeds ($V_{t\text{-}1}$, the central row), and angular velocity ($\Theta_t - \Theta_{t\text{-}1}$, the bottom column) for F2 (the left row) and F15 (the right row), when the time unit was 3 sec (T = 3).
