# Supplementary material for: Toward the Quantification of a Conceptual Framework for Movement Ecology Using Circular Statistical Modeling
Source: PLoS One. 2012 Nov 30;7(11):e50309. doi: 10.1371/journal.pone.0050309 (PMC3511459; doi:10.1371/journal.pone.0050309)
Supplement: Appendix S4 — Maximum likelihood estimates of the selected models for the 15 flight sections. (PDF) [file pone.0050309.s004.pdf]

Appendix-S4. Maximum likelihood estimates of the selected models for the 15 flight sections.

	Outward flight						Searching flight					Homeward flight			
Flight	F1	F2	F3	F4	F5	F6	F7	F8	F9	F10	F11	F12	F13	F14	F15
Length[1)]	4143.7	4322.6	4056.4	3796.4	3642.7	4432.0	2813.9	3928.7	3871.2	1803.1	3154.1	4964.6	5317.3	4963.5	4069.1
T[2)]	3	3	3	3	3	3	4	4	6	3	3	3	3	4	3
Note[3)]		α=arg	α=arg	λ=arg	λ=arg	λ=arg	λ=arg	λ=arg		$\lambda=c_1$			$\lambda=c_1$	$\lambda=c_1$	$\lambda=c_1$
Heading model															
Selected model	C-AR KJ	C-AR VM	C-AR VM	C-AR KJ	C-AR KJ	C-AR KJ	C-AR KJ	C-AR KJ	C-AR KJ	C-AR KJ	C-AR KJ	C-AR KJ	C-AR KJ	C-AR KJ	C-AR KJ
α	1.38	0.01	0.65	-	-	-	-	-	2.55	1.38	1.59	1.79	2.69	island	1.79
w	0.606	0.325	0.501	1	1	1	1	1	-0.024	0.020	0.241	0.567	0.209	0.207	0.093
κ	3.40	4.85	4.19	2.27	1.91	2.44	1.57	1.19	2.37	111.9	4.05	3.63	5.02	4.37	7.47
r	0.284	-	-	0.213	0.268	0.334	0.245	0.359	0.357	0.881	0.358	0.283	0.234	0.148	0.533
λ	-0.34	-	-	0.50	0.63	0.42	0.96	0.52	3.73	4.50	4.11	3.89	4.95	4.95	4.47
Speed model															
a	0.54	0.21	0.43	0.44	0.23	0.08	0.28	0.10	0.16	0	0.10	0.14	0.13	0.08	0
b	0.89	4.37	2.04	5.67	3.63	4.91	3.52	13.44	27.47	10.55	13.41	11.57	15.53	20.69	15.0
c	0.00	1.78	0.03	0.86	1.66	1.42	1.09	0.65	4.93	4.20	4.67	4.30	4.74	5.03	4.66
d	3.10	4.11	0	0	0	4.60	0	0	0	0	4.92	1.94	4.83	6.94	9.53
c_0	9.7	20.6	15.3	12.7	20.1	21.5	23.7	29.1	54.0	27.1	26.0	24.6	27.6	41.3	27.6
σ	2.49	3.02	2.08	2.50	2.34	1.99	4.78	5.47	11.13	6.18	4.71	3.12	4.24	6.79	5.18

1) The observed entire flight length, $|\overrightarrow{\mathbf{X}_0\mathbf{X}_n}|$.
2) Time unit.
3) $\alpha = \arg \overrightarrow{\mathbf{X}_0\mathbf{X}_n}$.
4) $\lambda = \arg \overrightarrow{\mathbf{X}_0\mathbf{X}_n}$.
