# Supplementary material for: Toward the Quantification of a Conceptual Framework for Movement Ecology Using Circular Statistical Modeling
Source: PLoS One. 2012 Nov 30;7(11):e50309. doi: 10.1371/journal.pone.0050309 (PMC3511459; doi:10.1371/journal.pone.0050309)
Supplement: Appendix S5 — Presence/absence of auto-correlations in residuals. (PDF) [file pone.0050309.s005.pdf]

Appendix-S5. Presence/absence of auto-correlations in residuals

For F2, auto-correlations in the residuals of C-AR(VM|$\alpha = \arg\overrightarrow{\mathbf{X}_0\mathbf{X}_n}$) were present at time unit T = 2 (A, $\hat{\rho}(1) = 0.28 > 1.96/\sqrt{249}$) but absent at T = 3 (B, $\hat{\rho}(1) = 0.12 < 1.96/\sqrt{165}$). For F15, auto-correlations in the residuals of the selected model, C-AR(KJ|$\lambda = \hat{c}_1$), were present at time unit T = 2 (C, $\hat{\rho}(1) = 0.23$) but absent at T = 3 (D, $\hat{\rho}(1) = 0.093$).

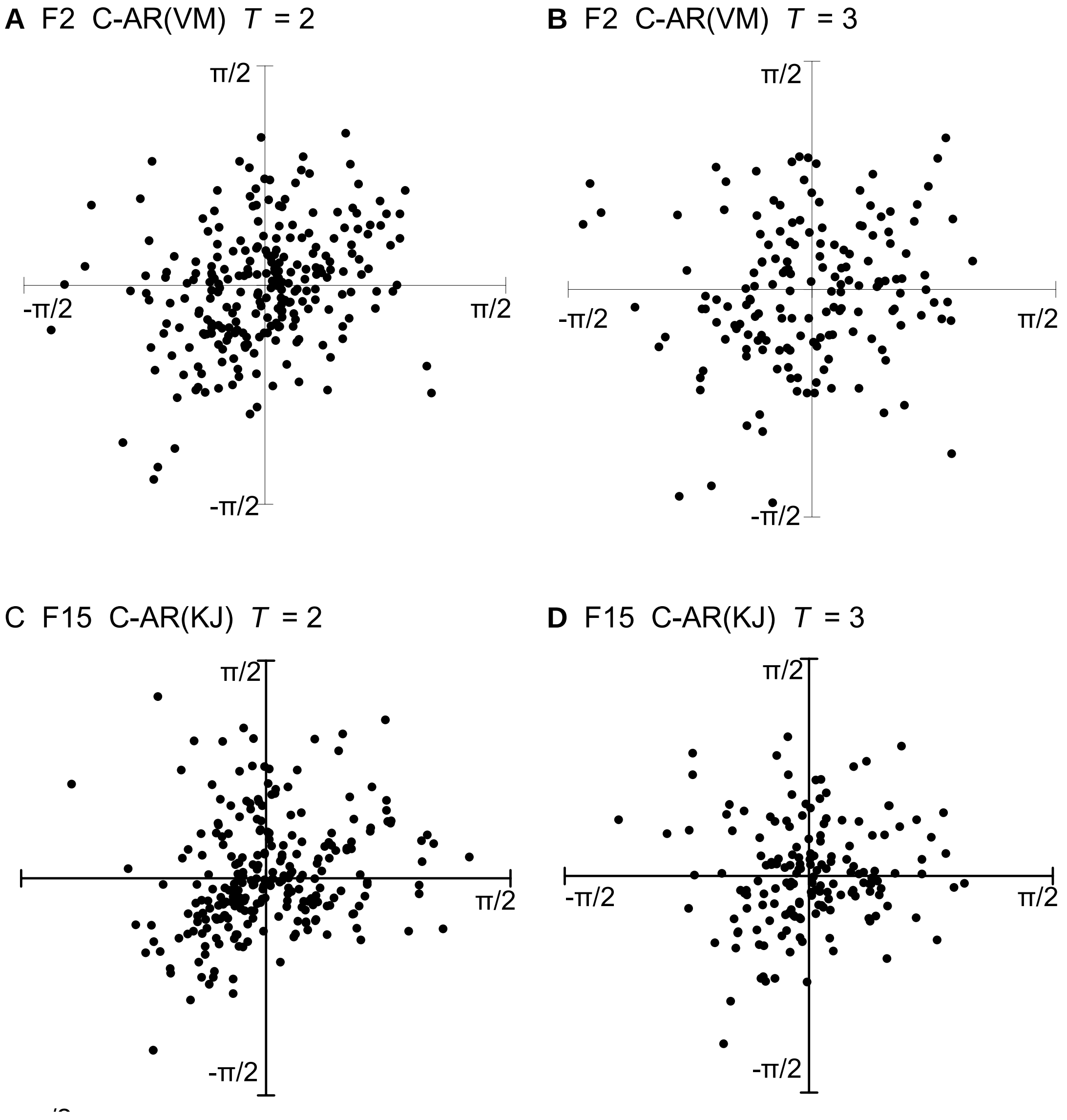

Figure S5. Scattering diagrams between the residuals. (A, B) The horizontal axes indicate $e_{t-1} = \Theta_{t-1} - M(\Theta_{t-2})$ and the vertical axes $e_t = \Theta_t - M(\Theta_{t-1})$. (C, D) The horizontal axes indicate $\overline{M}^{-1}(\Theta_t) - M(\Theta_{t-1})$ and the vertical axes $\overline{M}^{-1}(\Theta_{t-1}) - M(\Theta_{t-2})$. t = 2, 4, …, 249 in (A, C) and t = 2, 4, …, 165 in (B, D).
