# Supplementary material for: Toward the Quantification of a Conceptual Framework for Movement Ecology Using Circular Statistical Modeling
Source: PLoS One. 2012 Nov 30;7(11):e50309. doi: 10.1371/journal.pone.0050309 (PMC3511459; doi:10.1371/journal.pone.0050309)
Supplement: Appendix S6 — Correlations between the direction of heading and speed and predictions of the selected speed models. (PDF) [file pone.0050309.s006.pdf]

Appendix-S6. Correlations between heading directions and speeds and predictions of the selected speed models.

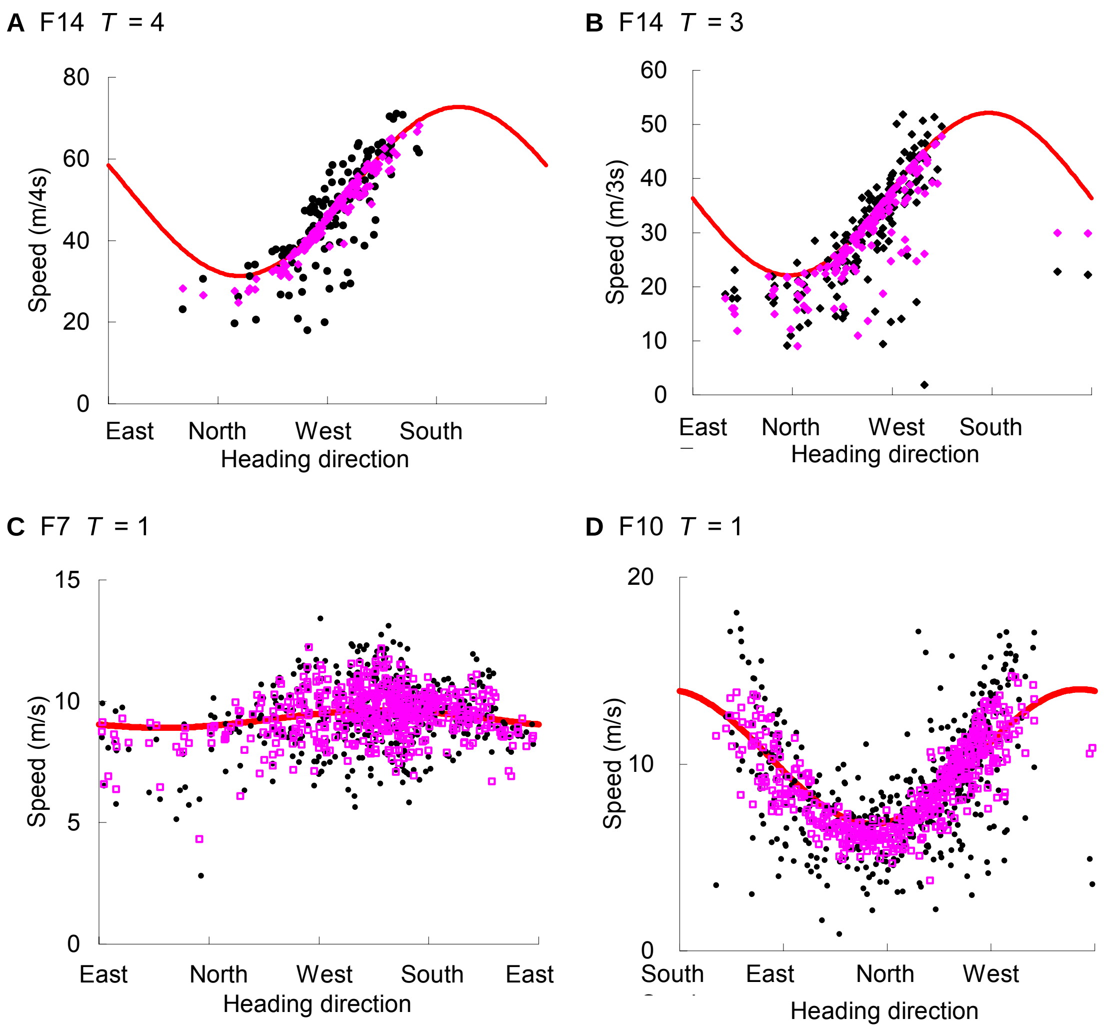

Figure S6. Black dots: Scattering diagram between observed heading directions Θ_t and observed speeds V_t. Pink dots: predicted speeds by the selected speed model plotted on each observed heading direction. Red curve: the expected speed as a function of heading directions when the previous speed was the mean over each flight section and angular velocity was 0.
