# Supplementary material for: Toward the Quantification of a Conceptual Framework for Movement Ecology Using Circular Statistical Modeling
Source: PLoS One. 2012 Nov 30;7(11):e50309. doi: 10.1371/journal.pone.0050309 (PMC3511459; doi:10.1371/journal.pone.0050309)
Supplement: Appendix S7 — Two examples of non-oriented trajectories produced by circular auto-regression. (PDF) [file pone.0050309.s007.pdf]

Appendix-S7. Two examples of non-oriented trajectories produced by circular auto-regression

A

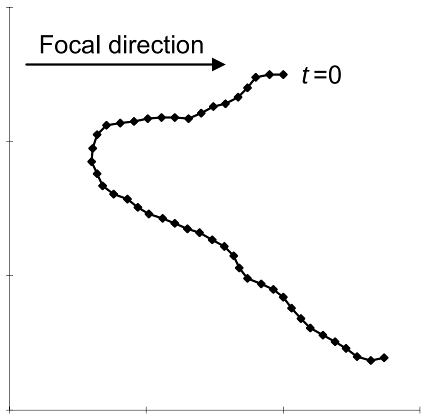

B

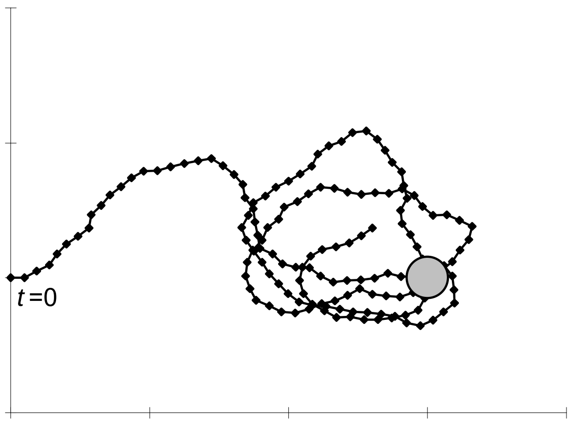

Figure S7. (A) Example of a simulated trajectory of C-AR(VM) of α = 0, *w* = 0.99, κ = 12. Starting toward the opposite direction to α, the trajectory is gradually changing toward the focal direction α. (B) An example of a simulated trajectory of C-AR(VM) with the focal point at (30, 0), *w* = 0.8, κ = 6. Starting from (0, 0), the trajectory is wandering around the focal point and draws looping. The speed is fixed at 1 and the grid unit is 10 throughout.
